# Distinct inflammation-related proteins associated with T cell immune recovery during chronic HIV-1 infection

**DOI:** 10.1080/22221751.2022.2150566

**Published:** 2022-12-20

**Authors:** Lin-Yu Wan, Hui-Huang Huang, Cheng Zhen, Si-Yuan Chen, Bing Song, Wen-Jing Cao, Li-Li Shen, Ming-Ju Zhou, Xiao-Chang Zhang, Ruonan Xu, Xing Fan, Ji-Yuan Zhang, Ming Shi, Chao Zhang, Yan-Mei Jiao, Jin-Wen Song, Fu-Sheng Wang

**Affiliations:** a The First Affiliated Hospital of USTC, Division of Life Sciences and Medicine, University of Science and Technology of China, Hefei, China; b Department of Infectious Diseases, the Fifth Medical Center of Chinese PLA General Hospital, National Clinical Research Center for Infectious Diseases, Beijing, China; c Department of Clinical Medicine, Bengbu Medical College, Bengbu, China; d Beijing Institute of Radiation Medicine, Beijing, China

**Keywords:** HIV-1, Inflammation, HIV-1 reservoir, CXCL9, CXCL10, CXCL11, T cell dysregulation

## Abstract

Chronic inflammation and T cell dysregulation persist in individuals infected with human immunodeficiency virus type 1 (HIV-1), even after successful antiretroviral treatment. The mechanism involved is not fully understood. Here, we used Olink proteomics to comprehensively analyze the aberrant inflammation-related proteins (IRPs) in chronic HIV-1-infected individuals, including in 24 treatment-naïve individuals, 33 immunological responders, and 38 immunological non-responders. T cell dysfunction was evaluated as T cell exhaustion, activation, and differentiation using flow cytometry. We identified a cluster of IRPs (cluster 7), including CXCL11, CXCL9, TNF, CXCL10, and IL18, which was closely associated with T cell dysregulation during chronic HIV-1 infection. Interestingly, IRPs in cluster 5, including ST1A1, CASP8, SIRT2, AXIN1, STAMBP, CD40, and IL7, were negatively correlated with the HIV-1 reservoir size. We also identified a combination of CDCP1, CXCL11, CST5, SLAMF1, TRANCE, and CD5, which may be useful for distinguishing immunological responders and immunological non-responders. In conclusion, the distinct inflammatory milieu is closely associated with immune restoration of T cells, and our results provide insight into immune dysregulation during chronic HIV-1 infection.

## Introduction

Human immunodeficiency virus type 1 (HIV-1) is a major global public health issue, as approximately 38 million individuals are infected with HIV worldwide [1]. The discovery and implementation of efficient, well-tolerated, combinational antiretroviral therapy (ART) has transformed these deadly viral infections into chronic manageable diseases. ART controls the replication of HIV-1, promotes the recovery of CD4 T cells, and improves the survival and quality of life of HIV-1-infected individuals.

Although ART can efficiently control viral replication, approximately 15–30% of HIV-1-infected individuals fail to achieve optimal recovery of CD4 T cell counts despite receiving ART [2]. These patients are referred to as immunological non-responders (INRs) and have a greater risk of serious non-acquired immunodeficiency syndrome (non-AIDS)-related events and mortality [3,4]. However, the precise mechanisms underlying this poor immune recovery are not well-understood. Older age, a low nadir CD4 T cell count, a longer duration of HIV-1 infection, and co-infection with hepatitis C virus are risk factors for suboptimal CD4 T cell count recovery [5–8]. To date, no additional treatment is available for INRs to efficiently restore CD4 T cell counts to normal levels. Therefore, studies are urgently needed to identify potential therapeutic targets for improving immune recovery in these patients. Residual low-grade inflammation persists even after ART treatment, particularly in INRs, and inflammation is associated with poor CD4 T cell recovery [9,10]. Previous studies demonstrated that the levels of sCD14, sCD163, and CXCL10 in the plasma are negatively correlated with CD4 T cell counts [11,12]. Additionally, the levels of sCD14, sCD163, CXCL10, CRP, and IL6 are related to HIV-1 disease progression [13–15]. These studies were performed using limited inflammatory markers; thus, inflammation-related proteins (IRPs) have not been comprehensively described in HIV-1-infected individuals, particularly in INRs.

Chronic HIV-1 infection leads to alterations in T cell subsets with altered maturation and differentiation profiles, in addition to increased levels of immune activation and exhaustion; these immunological scars persist even after ART treatment [16,17]. Higher levels of T cell activation and elevated expression of inflammatory markers, IL6 and sCD14, have been observed in INRs compared to those in immunological responders (IRs) [16]. IL6 and sCD14 are predictive of disease progression and associated with an increased risk of AIDS and death [15]. Additionally, IRPs are central to the homeostatic proliferation and differentiation of CD4 and CD8 T cells in HIV-1 infection [18]. For example, IL7 and IL15 are the principal regulators of CD4 and CD8 T cell homeostasis [19,20]. CXCL10 drives CD4^+^ T_N_ cells towards Th1 cell polarization [21]; the levels of CXCL10 are positively correlated with activation of CD4 and CD8 T cells, regardless of whether the patients have been subjected to ART [22]. However, the association between IRPs and T cell dysregulation is not well-understood.

In this study, we assessed the systemic levels of 92 IRPs in HIV-1-infected individuals and explored the association between IRPs and HIV-1 clinical parameters and T cell dysregulation, including T cell differentiation, activation, and exhaustion. We observed significant associations between IRPs in cluster 7 and HIV-1 disease progression. We also identified several IRPs in cluster 5 that were negatively associated with HIV-1 DNA levels in ART-treated individuals. Particularly, we identified a combination of IRPs that can be used to distinguish between INRs and IRs. This comprehensive analysis of chronic inflammation in HIV-1-infected individuals may provide potential targets for improving the prognosis of these patients.

## Materials and methods

### Patients and samples

Ninety-five HIV-1-infected individuals were enrolled from the Fifth Medical Center of Chinese PLA General Hospital. Among these patients, 24 were treatment-naïve HIV-1-infected individuals (TNs), 33 were IRs, and 38 were INRs. IRs and INRs were defined as having CD4 T cell counts above and below 350 cells/μL, respectively; individuals in both groups had received ART for more than two years and had plasma HIV-1 viral loads below 80 copies/mL. Twenty healthy controls (HCs) were also included in this study. The exclusion criteria were as follows: pregnancy, AIDS-related diseases, chronic hepatitis B or hepatitis C infection, ongoing immunosuppressive therapy, and moribund status. Plasma samples were collected and stored at −80°C until analysis. Peripheral blood mononuclear cells (PBMCs) were isolated using Ficoll‒Hypaque (MD Pacific Biotechnology, Tianjin, China) density gradient centrifugation.

### Flow cytometry

PBMCs were incubated in Zombie NIR™ Fixable Viability Kit (BioLegend, San Diego, CA, USA) at 4°C for 20 min and then washed. PBMCs were stained with the following human antibodies: CD3-BV650 (clone OKT3), CD27-BV421 (clone M-T271), CD45RA-BV711 (clone HI100), HLA-DR-BV605 (clone L243), CD38-PE-Cy7 (clone HB-7), and PD-1-BV510 (clone EH12.2H7), purchased from BioLegend, and CD8-BUV737 (clone SK1) and CCR7-BB700 (clone 3D12), purchased from BD Biosciences (Franklin Lakes, NJ, USA). Events were detected on a BD FACSymphony^TM^ A5 flow cytometer and the data were analyzed using FlowJo software V10 (Tree Star, Ashland, OR, USA).

Dimensionality reduction was performed using the FlowJo plugin UMAP version 2.2. The DownSample version 3.0.0 plugin and concatenation tool were used to visualize multiparametric data from up to 320,000 T cells.

### Proteomic profiling of soluble factors in plasma

Plasma samples from the cohort were quantified using the Olink multiplex proximity extension assay (PEA) inflammation panel that included 92 IRPs (Olink Bioscience AB, Uppsala, Sweden). PEA uses pairs of oligonucleotide-labeled antibodies to bind to their respective target proteins. When two antibodies are in close proximity, a new polymerase chain reaction (PCR) target sequence is formed through a proximity-dependent DNA polymerization reaction. The resulting sequence was detected and quantified using standard real-time PCR. Olink-generated data were presented as normalized protein expression values, which showed comparable distributions as the log_2_-transformed protein concentrations. Among our 115 samples, two samples did not pass quality control or deviated from the target populations and thus were excluded from analysis. More than 90% of the values for 18 proteins were below the lower limit of detection; therefore, these proteins were also excluded from further analysis. Seventy-four IRPs from 113 individuals were evaluated in this study. Further information on the Olink assay is available at http://www.olink.com. Venn diagrams were constructed using an online system (http://www.ehbio.com/test/venn).

### Quantification of HIV-1 DNA and RNA

Total cellular DNA and RNA were extracted from fresh PBMCs using a Qiagen QIAsymphony DNA Mini Kit (Hilden, Germany) and HiPure Total RNA Plus Mini Kit (Magen, Guangzhou, China), respectively. A fluorescence-based real-time SUPBIO HIV-1 Quantitative Detection Kit (SUPBIO, Guangzhou, China) was used to quantify the HIV-1 DNA and RNA. The detection range was 10‒5 × 10^6^ copies per 10^6^ PBMCs.

### Statistical analysis

GraphPad Prism version 8.0 software (GraphPad, Inc., San Diego, CA, USA), SPSS version 22.0 software (SPSS, Inc., Chicago, IL, USA), and R Studio version 3.6.4 software (RStudio, Boston, MA, USA) were used to analyze the data. Gene Ontology term enrichment analysis was performed using the DAVID online tool (https://david.ncifcrf.gov/). Continuous variables are expressed as the median interquartile range, and categorical variables are expressed as the count (%). The χ^2^ test was used to compare categorical data between groups. Mann–Whitney U test (between two groups) and Kruskal–Wallis test (for more than two groups) were used to compare continuous data. The *P*-values were adjusted for multiple comparisons using the Benjamini‒Hochberg method. Correlations were identified using Spearman rank correlation test between two continuous variables. *P*-values < 0.05 were considered to indicate that the results were statistically significant.

## Results

### Characteristics of study participants

**Figure 1.**
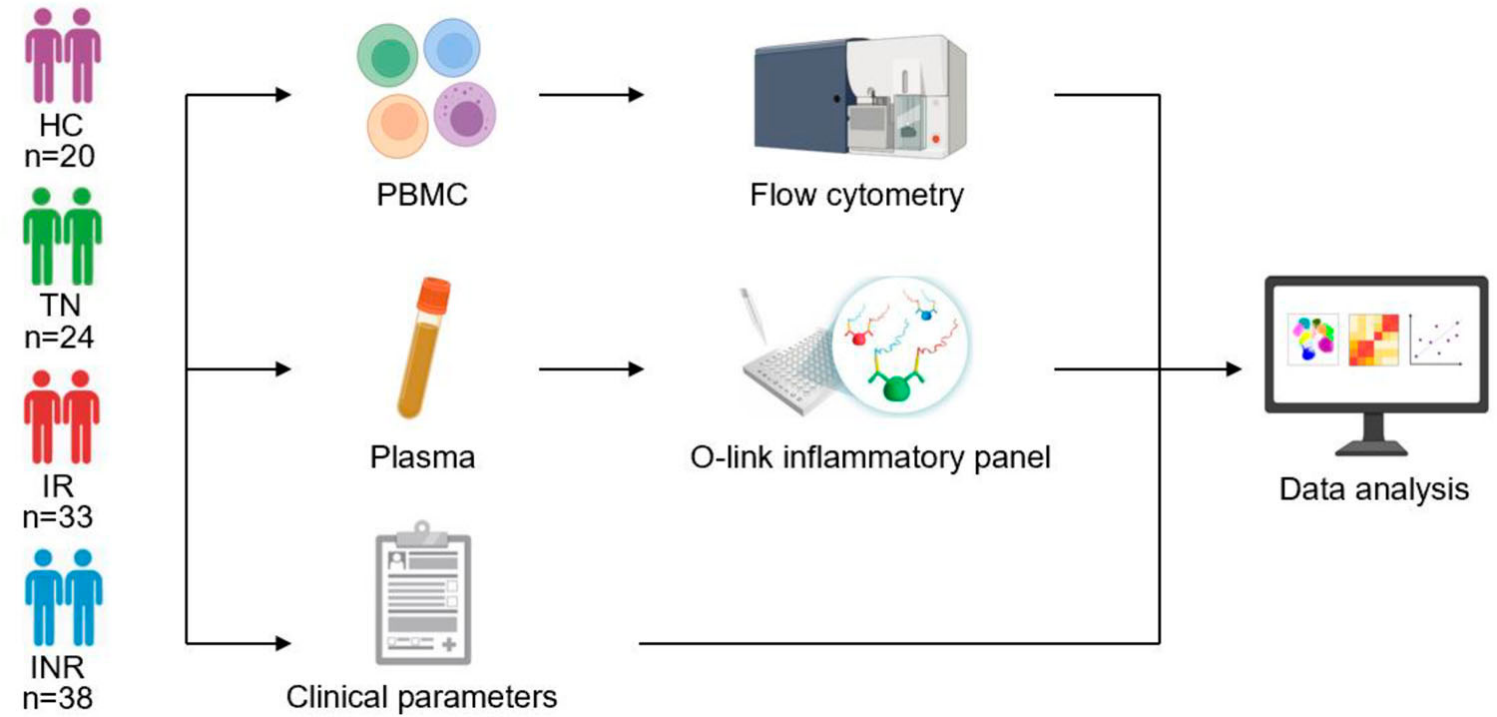
Study design. Peripheral blood mononuclear cells (PBMCs) and plasma were collected from healthy controls (HCs), treatmentnaïve individuals (TNs), immunological responders (IRs), and immunological non-responders (INRs). Flow cytometry was used to evaluate T cell dysfunction, including T cell exhaustion, activation, and differentiation. The O-link inflammatory panel was used to measure 92 inflammation-related proteins (IRPs). The clinical parameters of the enrolled participants were also obtained. The association between IRPs and HIV-1 clinical parameters and T cell dysregulation were analyzed.

To investigate the characteristics and clinical implications of plasma IRPs in HIV-1 infected individuals, four groups were included in this study: 20 HCs, 24 TNs, 33 IRs and 38 INRs (Figure 1). The characteristics of the participants are summarized in Table 1. The INR group was older than the IR group (median, 43.5 vs. 31 years, *P* < 0.0001). The median CD4 T cell counts were 196.0, 656.0, 392.0, and 768.0 cells/μL in INRs, IRs, TNs, and HCs, respectively. In addition, the baseline CD4 T cell count in the INR group was lower than that in the IR group (median, 60.5 vs. 344.0 cells/μL, *P* < 0.0001). The duration and regimen of ART did not differ significantly between IRs and INRs.

**Table 1.** Clinical characteristics of study subjects.

	HC	TN	IR	INR	*P* value
*n*	20	24	33	38	
Age (years)	30.0 (27.3–31.8)	28.0 (26.0–34.0)	31.0 (27.5–34.5)	43.5 (36.8–49.3)	<0.0001^a^
Gender, male, n (%)	15 (75%)	23 (96%)	33 (100%)	35 (92%)	<0.01 ^b^
BMI, kg/m^2^	22.1 (19.7–25.3)	21.7 (17.8–24.4)	23.2 (20.9–25.6)	22.5 (19.7–24.2)	0.3290 ^a^
CD4 T cell counts (cells/μl)	768.0 (525.5–932.5)	392.0 (150.8–652.3)	656.0 (541.5–807.0)	196.0 (159.3–238.3)	<0.0001 ^a^
CD4 T cell counts at baseline (cells/μl)	NA	NA	344.0 (201.0–449.5)	60.5 (17.8–121.3)	<0.0001 ^c^
CD8 T cell counts (cells/μl)	622.0 (513.0–1032.0)	1043.0 (820.5–1235.0)	922.0 (631.5–1100.0)	607.5 (412.4–818.8)	<0.01 ^a^
CD4/CD8 ratio	1.1 (0.9–1.4)	0.4 (0.2–0.5)	0.8 (0.6–1.0)	0.3 (0.2–0.5)	<0.0001 ^a^
Time on ART (months)	NA	NA	48.8 (39.0–63.9)	45.4 (33.6–54.4)	0.1794 ^c^
ART regimen, n (%)	NA	NA			0.1237 ^b^
NRTIs + NNRTIs			31 (94%)	38 (100%)	
NRTIs + PIs			2 (6%)	0 (0%)	
Viral load (copies/ml)	NA	17,650 (5,480–39,625)	<LDL	<LDL	NA
Viral load at baseline (copies/ml)	NA	NA	116,150 (46,175–595,750)	207,500 (21,525–608750)	0.7574 ^c^

Continuous variables are expressed as the median (interquartile range, IQR) and categorical variables are expressed as the number of cases (%). HC, healthy control; TN, treatment-naïve HIV-1-infected individual; IR, immunological responder; INR, immunological non-responder; BMI, body mass index; ART, antiretroviral therapy; NA, not available; LDL, below lower detection limit; NRTIs, nucleoside reverse transcriptase inhibitors; NNRTIs, non-nucleoside reverse transcriptase inhibitors; PIs, protease inhibitors; ^a^Kruskal-Wallis test; ^b^*χ*^2^ test; ^c^Mann-Whitney *U* test.

### T cell differentiation in HIV-1-infected individuals

**Figure 2.**
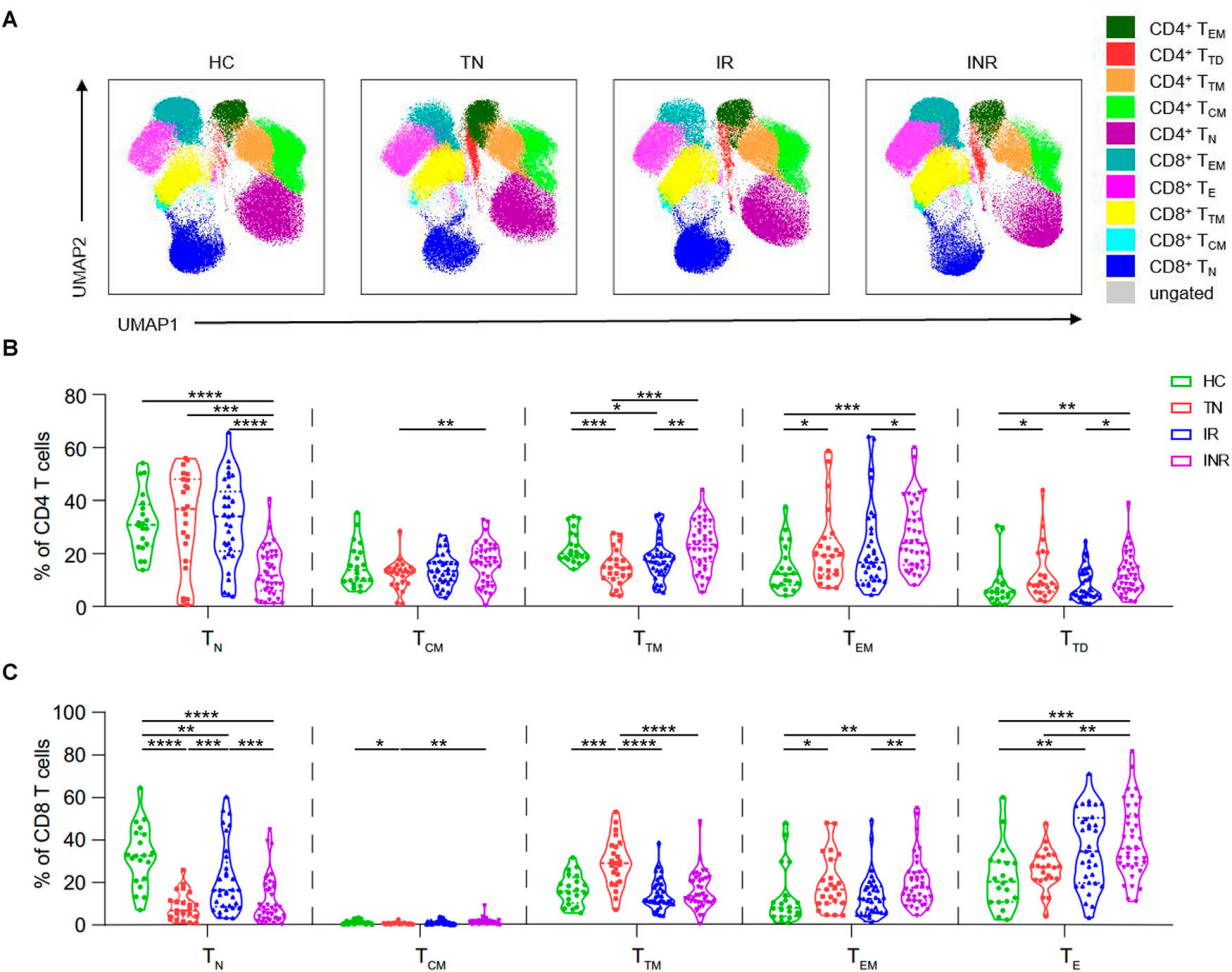
T cell subsets in HIV-1-infected individuals. CD45RA, CD27, and CCR7 expression in peripheral blood mononuclear cells (PBMCs) was determined using flow cytometry. T cell subsets were defined as follows: naïve (T_N_; CD45RA^+^ CD27^+^ CCR7^+^), central memory (T_CM_; CD45RA^−^ CD27^+^ CCR7^+^), transitional memory (T_TM_; CD45RA^−^ CD27^+^ CCR7^−^), effector memory (T_EM_; CD45RA^−^ CD27^−^ CCR7^−^), and terminal differentiated/effector (T_TD_/T_E_; CD45RA^+^ CD27^+^ CCR7^−^). (A) Uniform manifold approximation and projection (UMAP) analysis of T cell subsets in healthy controls (HCs), treatment-naïve individuals (TNs), immunological responders (IRs), and immunological non-responders (INRs). Each point on the high-dimensional mapping represents an individual cell, and the UMAP plots show the cell populations in different colours. (B–C) Proportions of (B) CD4 and (C) CD8 T cell subsets in HCs (*n* = 20), TNs (*n* = 23), IRs (*n* = 33), and INRs (*n* = 38). Data are expressed as the median (interquartile range, IQR). Each dot represents a participant. Statistical significance between two groups was determined using non-parametric unpaired Mann–Whitney *U* test. *P < 0.05, **P < 0.01, ***P < 0.001, ****P < 0.0001.

We assessed the CD4 and CD8 T cell subset composition among HIV-1-infected individuals using flow cytometric analysis. T cell subsets were identified based on CD45RA, CD27, and CCR7 expression (Figure S1) [23]. The distribution of T cell subsets and expression of CD8, CD45RA, CD27, and CCR7 in PBMCs from the four groups were visualized using uniform manifold approximation and projection (UMAP) (Figure 2(A), Figure S2). In CD4 T cells, the frequency of transitional memory (T_TM_) cells decreased (*P* = 0.0002), but the frequencies of effector memory (T_EM_) and terminally differentiated (T_TD_) cells increased in TNs compared to those in HCs (*P* = 0.0493 and *P* = 0.0301, respectively) (Figure 2(B)). In CD8 T cells, the frequencies of naïve (T_N_) and central memory (T_CM_) cells decreased (*P* < 0.0001 and *P* = 0.0464, respectively), whereas the frequencies of T_TM_ and T_EM_ cells increased in TNs compared with those in HCs (*P* = 0.0001 and *P* = 0.0177, respectively) (Figure 2(C)). Despite long-term suppression of HIV-1 replication with ART, the distribution of T cell subsets was not fully restored. In INRs, the frequencies of CD4^+^ T_EM_ (INRs vs. IRs, *P* = 0.0319; INRs vs. HCs, *P* = 0.0002), CD8^+^ T_EM_ (INRs vs. IRs, *P* = 0.0042; INRs vs. HCs, *P* = 0.0015), and CD4^+^ T_TD_ cells (INRs vs. IRs, *P* = 0.0228; INRs vs. HCs, *P* = 0.0044) increased, and the frequencies of CD4^+^ T_N_ (INRs vs. IRs, *P* < 0.0001; INRs vs. HCs, *P* < 0.0001) and CD8^+^ T_N_ cells (INRs vs. IRs, *P* = 0.0008; INRs vs. HCs, *P* < 0.0001) decreased compared to those in IRs and HCs (Figure 2(B,C)).

We also evaluated the expression of activation and exhaustion markers on T cells (Figures S2, S3). We found that T cell activation and exhaustion were significantly increased in TNs compared to that in HCs, and ART did not fully restore normal function, particularly in INRs. The frequencies of HLA-DR^+^, CD38^+^, and HLA-DR ^+ ^CD38^+^ CD4 T cells were higher in INRs than in IRs (*P* < 0.0001, *P* = 0.0133, and *P* = 0.0001 respectively). The frequencies of PD-1^+^ CD4 (INRs vs. IRs, *P* < 0.0001; INRs vs. HCs, *P* < 0.0001) and PD-1^+^ CD8 (INRs vs. IRs, *P* = 0.0162; INRs vs. HCs, *P* = 0.0008) T cells were higher in INRs than in IRs and HCs.

### Soluble IRPs in plasma

**Figure 3.**
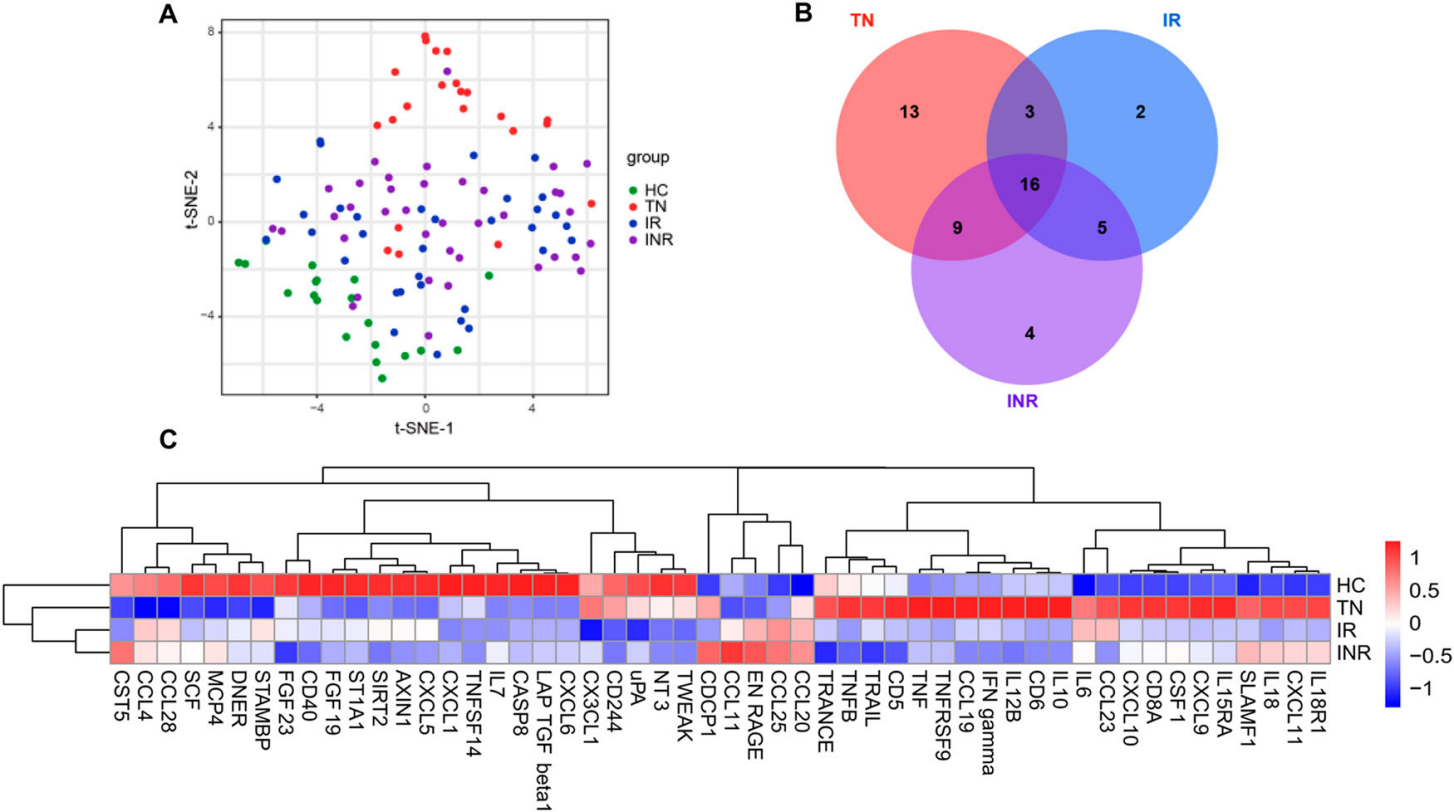
Altered inflammation-related proteins in plasma from HIV-1-infected individuals. (A) t-Distributed stochastic neighbour embedding visualization of individuals with different categories based on inflammation-related proteins (IRPs) in plasma detected using the Olink platform. (B) Venn diagram showing the number of differentially expressed IRPs in different combinations [treatment-naïve individuals (TNs) vs. healthy controls (HCs), immunological responders (IRs) vs. HCs, immunological non-responders (INRs) vs. HCs]. (C) Heatmap of differentially expressed IRPs.

We measured 92 IRPs using the Olink multiplex inflammation panel to investigate the clinical implications of plasma IRPs in HIV-1-infected individuals. The t-SNE analysis based on the expression of IRPs clearly separated HCs, TNs, and HIV-1-infected individuals with ART (IRs plus INRs) (Figure 3(A)). Fifty-two IRPs significantly differed between HIV-1-infected individuals (TNs, IRs, and INRs) and HCs (Figure 3(B)); 41 IRPs were significantly different between TNs and HCs (Table S1), 26 between IRs and HCs (Table S2), and 34 between INRs and HCs (Table S3). In addition, 27 IRPs were significantly different between IRs and TNs (Table S4), 27 between INRs and TNs (Table S5), and 9 between IRs and INRs (Table S6). The differential expression of these IRPs was visualized using heatmap (Figure 3(C)). The IRP profiles significantly differed between HIV-1-infected individuals and HCs, particularly TNs.

### IRPs correlate with HIV-1 disease progression in TNs

We evaluated the pairwise correlation between 41 differentially expressed IRPs in TNs and HCs. The IRPs were separated into seven clusters (1‒7, from top to bottom) based on their correlation coefficients (Figure 3(A)). Combined with the results shown in Figure 3(C), HIV-1 infection led to decreased levels of IRPs in clusters 1, 4, and 5 and increased levels of IRPs, except for that of CST5, in clusters 6 and 7.

**Figure 4.**
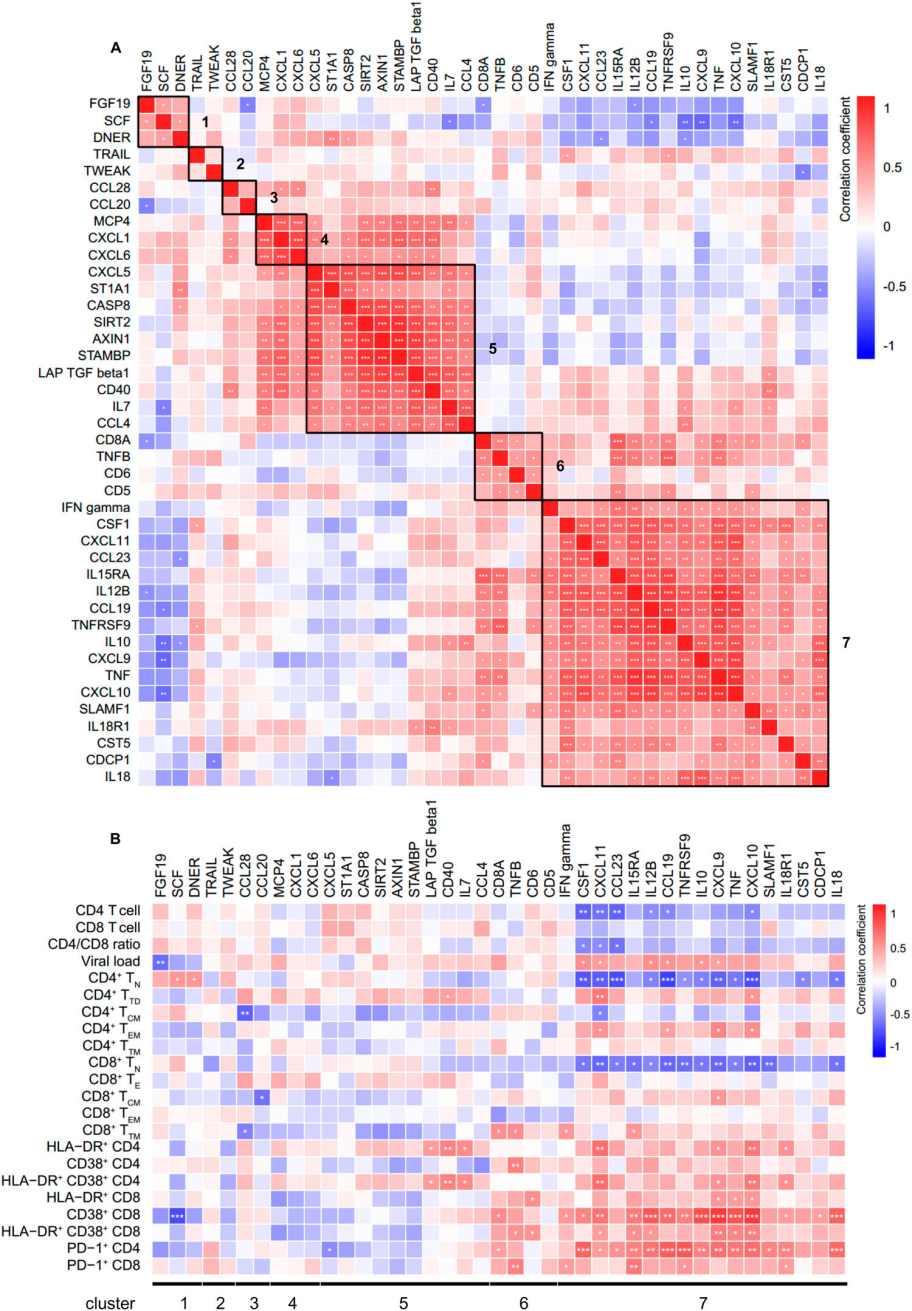
Association between inflammation-related proteins with clinical and immunological variables in treatment-naïve HIV-1-infected individuals. Differentially expressed IRPs between treatment-naïve individuals (TNs) and healthy controls (HCs) were determined. (A) Correlation among inflammation-related proteins (IRPs) is displayed using Spearman’s correlation matrix. Based on the correlation between the IRPs, these proteins were separated into seven clusters (1–7, from top to bottom). The clusters are highlighted in heatmap black boxes. (B) Heatmap showing Spearman correlations between differentially expressed proteins and clinical and immunological variables. Red and blue indicate positive and negative associations, respectively. *P < 0.05, **P < 0.01, ***P < 0.001.

We further evaluated the correlation between IRPs and clinical and immunological variables in TNs; IRPs in cluster 7 were closely related to HIV-1 disease progression (Figure 4(B)). Among the IRPs in cluster 7, CD4 T cell count was negatively correlated with the levels of CSF1, CXCL11, CCL23, IL12B, CCL19, and CXCL10. The HIV-1 viral load was positively correlated with CSF1, CXCL11, IL12B, CCL19, IL10, and CXCL9 levels. The frequencies of CD4^+^ T_N_ and CD8^+^ T_N_ cells were negatively correlated with the levels of CSF1, CXCL11, CCL23, IL12B, CCL19, TNFRSF9, IL10, CXCL9, TNF, CXCL10, and IL18. The frequency of HLA-DR^+^ CD38^+^ CD4 T cells was positively correlated with the levels of CXCL11, CXCL9, CXCL10, and IL18R1. The frequency of HLA-DR^+^ CD38^+^ CD8 T cells was positively correlated with the levels of CXCL11, IL15RA, IL12B, CXCL9, TNF, and CXCL10. The frequency of PD-1^+^ CD4 T cells was positively correlated with the levels of CSF1, CXCL11, CCL23, IL15RA, IL12B, CCL19, TNFRSF9, IL10, CXCL9, TNF, CXCL10, SLAMF1, IL18R1, and IL18. The frequency of PD-1^+^ CD8 T cells was positively correlated with the levels of IFN-γ, IL15RA, TNFRSF9, and IL18R1.

We also performed GO analysis of the 17 IRPs in cluster 7 and found that the most represented categories were “inflammatory response,” “positive regulation of NIK/NF-kappaB signalling,” “chemokine-mediated signalling pathway,” “cellular response to lipopolysaccharide,” and “positive regulation of interferon-gamma production,” suggesting that these biological processes are altered during HIV-1 infection (Table S7).

### IRPs correlate with immune recovery in patients on ART

**Figure 5.**
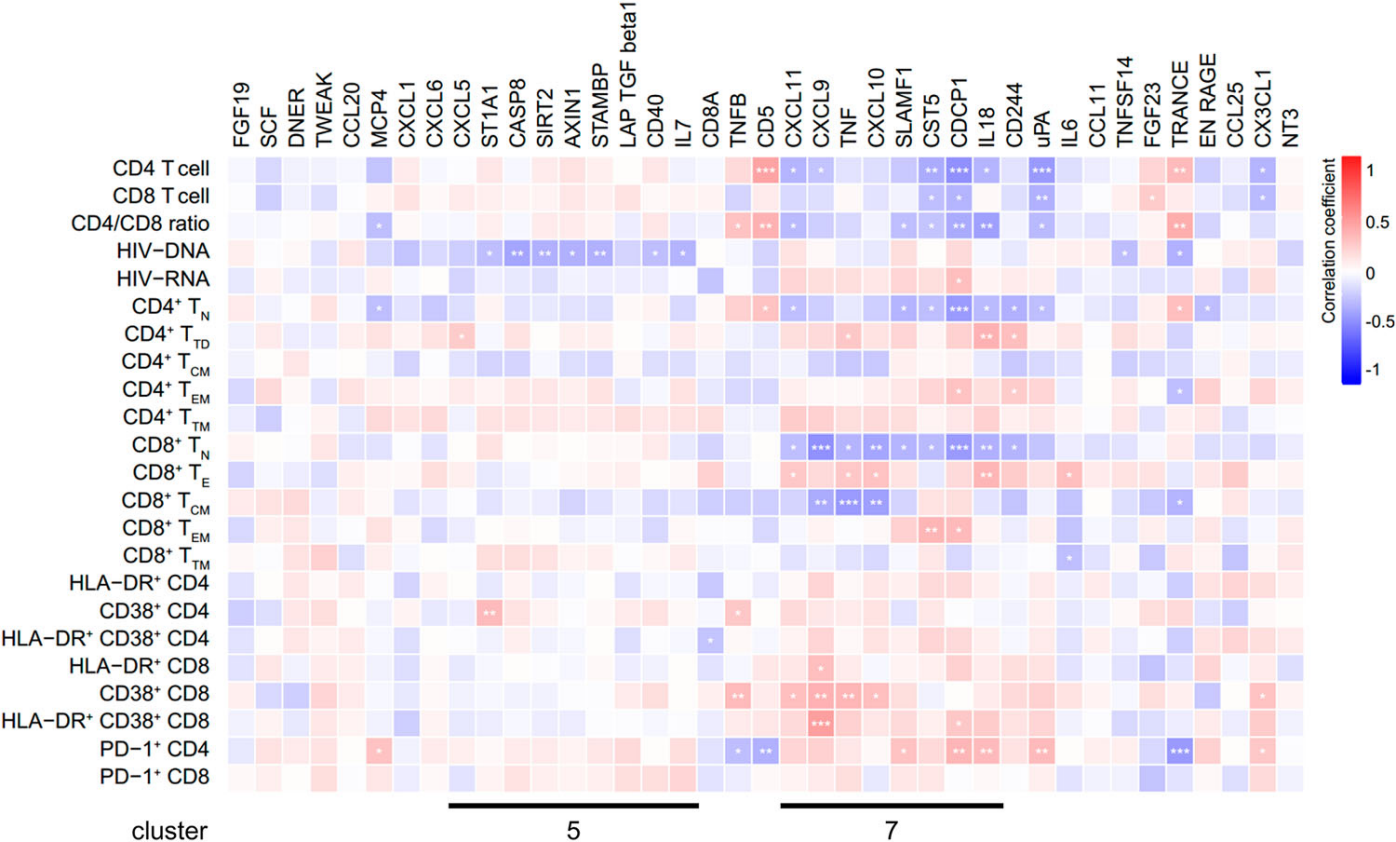
Associations between inflammation-related proteins with clinical and immunological variables in patients on ART. Differentially expressed inflammation-related proteins (IRPs) between antiretroviral therapy (ART)-treated HIV-1-infected individuals and healthy controls (HCs) were determined. The heatmap shows the Spearman correlations between the differentially expressed proteins and clinical and immunological variables. Red and blue indicate positive and negative associations, respectively. *P < 0.05, **P < 0.01, ***P < 0.001.

We found that the levels of CXCL11, CXCL9, TNF, CXCL10, SLAMF1, CST5, CDCP1, and IL18 in cluster 7 remained abnormal after ART (Figure S4). Analysis of the correlation between IRPs and clinical and immunological variables in patients on ART revealed that the abnormal IRPs in cluster 7 were closely related to clinical parameters (Figure 5). In patients on ART, CD4 T cell counts and the CD4/CD8 ratio were negatively correlated with CXCL11 levels. The frequencies of CD4^+^ T_N_ and CD8^+^ T_N_ cells were negatively correlated with levels of CXCL11 and IL18, the frequency of HLA-DR^+^ CD38^+^ CD8 T cells was positively correlated with the level of CXCL9, and the frequency of PD-1^+^ CD4 T cells was positively correlated with levels of SLAMF1 and IL18. Furthermore, we measured HIV-1 DNA and cell-associated unspliced RNA in patients on ART. HIV-1 DNA levels were positively correlated with HIV-1 RNA levels in ART-treated patients (IRs plus INRs) (*r* = 0.2556, *P* = 0.0314) and IRs (*r* = 0.4206, *P* = 0.0148), whereas no correlation was observed in INRs (Figure S5). A significant negative association was detected between HIV-1 DNA and the levels of ST1A1, CASP8, SIRT2, AXIN1, STAMBP, CD40, and IL7 in cluster 5 (Figure 5). CDCP1 levels were positively correlated with HIV-1 RNA levels (Figure 5). Interestingly, evaluation of the correlation between IRPs and clinical and immunological variables in IRs and INRs showed that most IRPs in cluster 5, including CXCL5, ST1A1, CASP8, SIRT2, AXIN1, STAMBP, and IL7, were negatively correlated with HIV-1 DNA levels in IRs but not with those in INRs (Figure S6A, S6B).

**Figure 6.**
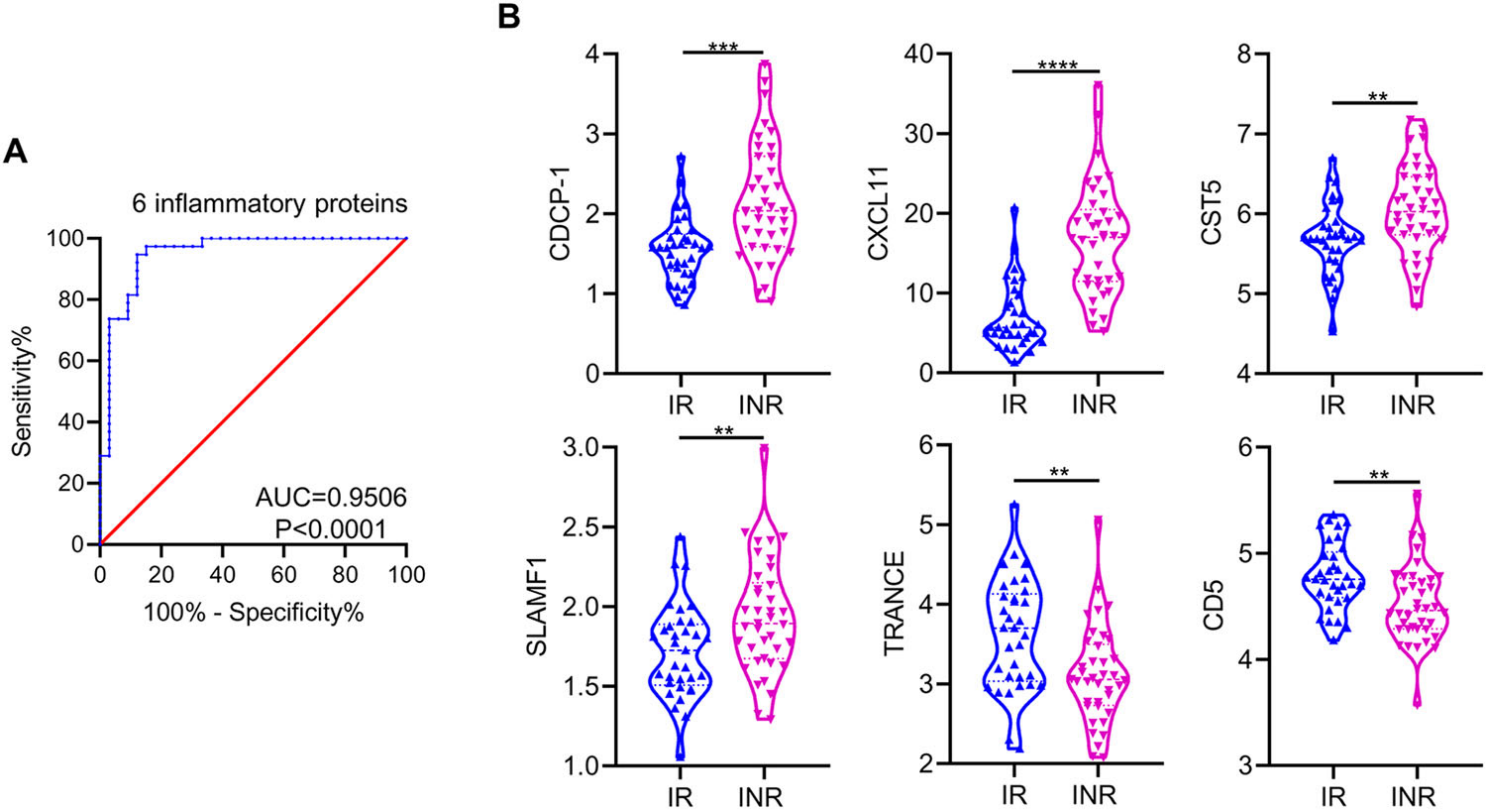
Inflammation-related proteins for differentiating immunological responders and immunological non-responders in plasma. (A) Panel of six differentially expressed inflammation-related proteins (IRPs), including CDCP-1, CXCL11, CST5, SLAMF1, TRANCE, and CD5, was used to distinguish immunological non-responders (INRs) from immunological responders (IRs). (B) Expression levels of CDCP-1, CXCL11, CST5, SLAMF1, TRANCE, and CD5 in IRs and INRs. Data are expressed as the median (interquartile range, IQR). Statistical significance between two groups was determined by non-parametric, unpaired Mann–Whitney *U* test. AUC, area under the curve. *P < 0.05, **P < 0.01, ***P < 0.001, ****P < 0.0001.

To identify IRPs that could distinguish INRs from IRs, nine significantly differentially expressed proteins between IRs and INRs, including CDCP1, OPG, CD5, uPA, CXCL11, CST5, SLAMF1, TRANCE, and Flt3L (Table S6), were examined using multivariable linear regression. Six IRPs, including CDCP1, CXCL11, CST5, SLAMF1, TRANCE, and CD5, were significant factors (Table S8). Receiver operating characteristic curve analysis indicated that these six proteins had a differentiated value of 95.06% (*P* < 0.0001) between IRs and INRs, and the model was verified by leave-one-out cross-validation (Figure 6(A)). Among the six IRPs, the levels of CDCP1, CXCL11, CST5, and SLAMF1 were higher in INRs than in IRs and the levels of TRANCE and CD5 were lower in INRs than in IRs (Figure 6(B)). We also analyzed the expression of these six IRPs among TNs, which were categorized into two groups based on low and high CD4 T cell counts and viral loads, respectively. These IRPs did not differ between the two groups. (Figure S7).

Furthermore, we performed GO analysis of 26 differentially expressed IRPs between IRs and HCs and 34 differentially expressed IRPs between INRs and HCs. Among the top 10 biological processes, the “cellular response to LPS” was specific to INRs (Tables S79 and S10). Consistently, a previous study showed that the plasma levels of lipopolysaccharide (LPS) are higher in INRs than in IRs [24]. LPS is the major component of the gram-negative bacterial outer membrane and is highly antigenic. LPS is responsible for chronic immune activation and inflammation during HIV-1 infection [25]**.**

## Discussion

**Figure 7.**
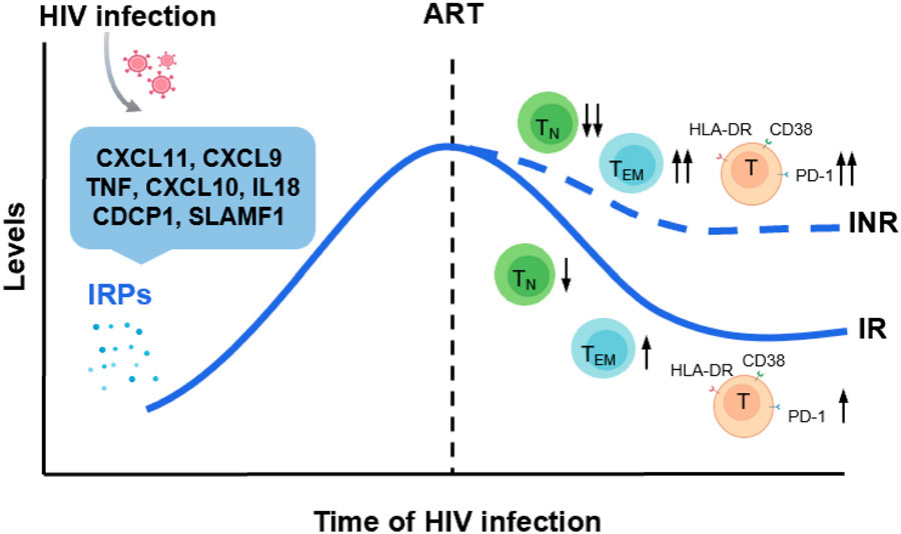
Significantly altered inflammation-related proteins and their relationship with immune recovery in HIV-1 infected individuals. HIV-1 infection leads to the upregulation of a cluster inflammation-related proteins (IRPs) (cluster 7), including CXCL11, CXCL9, TNF, CXCL10, IL18, CDCP1, and SLAMF1. These IRPs remained abnormal despite antiretroviral therapy (ART), particularly in immunological non-responders (INRs). Importantly, these IRPs in cluster 7 were closely associated with T cell differentiation, activation, and exhaustion.

Despite the successful control of HIV-1 viremia achieved using ART, HIV-1-infected individuals, particularly the INR population, still experience a higher incidence of non-AIDS-related events, which is partially attributed to residual chronic inflammation and immune activation. In this study, we systematically analyzed IRPs in HIV-1-infected individuals with various disease statuses and analyzed the association of these IRPs with T cell dysfunction and HIV-1 disease progression. We found that HIV-1 infection altered T cell differentiation, activation, and exhaustion. Abnormal expression of IRPs is closely related to HIV-1 disease progression and T cell dysfunction (Figure 7).

In our study, INRs were older and had lower baseline CD4 T cell counts compared to those of IRs, which is in consistent with previous studies showing that the older age and low baseline CD4 T cell counts are associated with poor immune recovery in HIV-1-infected individuals [5,6,8]. Previous studies showed that T_N_ cells decreased with advanced age [26,27] and exhibited proliferation and effector differentiation defects, further contributing to T cell dysfunction and activation among older patients [27,28].

HIV-1 infection-induced T cell dysfunction is not fully recovered by ART treatment, particularly in INRs. Consistent with the results of previous studies [29–31], we observed decreased frequencies of CD4^+^ T_N_ and CD8^+^ T_N_ cells, as well as increased frequencies of CD4^+^ T_EM_ and CD8^+^ T_EM_ cells, in INRs compared to those in IRs and HCs. Decreased frequencies of T_N_ cells may be related to reduced thymic output and the differentiation of T cells into memory and effector T cells [2,32]. T_EM_ cells can migrate to inflamed peripheral tissues and exert effector functions; however, the increased frequencies of T_EM_ cells may be related to over-activation and accelerated exhaustion in INRs. In addition, compared to that in HCs and IRs, T cell activation and exhaustion were higher in INRs [16,29]. The mechanisms underlying immune activation are complex. Translocation of intestinal microbes into the bloodstream is one cause of systemic immune activation during HIV-1 infection [33]. LPS, an indicator of microbial translocation, is significantly increased during HIV-1 infection and is higher in INRs than in IRs [24,34,35]. In addition, regulatory T cells play important roles in inhibiting T cell activation and proliferation. However, regulatory T cells are decreased in HIV-1-infected individuals and may lead to abnormal immune activation [36]. Persistent activation of T cells may also lead to T cell exhaustion.

Inflammation is a hallmark of HIV-1 infection, but the association between IRPs and T cell dysregulation is not fully understood. Therefore, we evaluated the levels of 92 IRPs and found that CXCL11, CXCL9, TNF, CXCL10, SLAMF1, and IL18 in cluster 7 were closely associated with T cell differentiation, T cell activation and exhaustion, and disease progression in TNs. Some differentially expressed IRPs between HCs and TNs remained abnormal after ART, specifically the proteins in cluster 7. Particularly, CXCL9, CXCL10, and CXCL11 in cluster 7 were closely associated with disease progression and immune disorders, irrespective of whether the patients had been treated with ART. CXCL9, CXCL10, and CXCL11 are IFN-γ-induced chemokines secreted by monocytes, endothelial cells, and fibroblasts and interact with cell surface CXCR3 [37]. The interaction of these ligands with CXCR3 affects both the migration of T cells into inflamed regions and their polarization into effector T cells [38]. In addition, CXCL9, CXCL10, and CXCL11 play important roles in regulating the differentiation of T_N_ cells to Th1 cells and in immune activation [39]. Th1 cells produce IFN-γ, TNF, and IL2 and promote immune reactivity by stimulating cytotoxic T cells, natural killer cells, natural killer T cells, and macrophages [40]. IL18, secreted primarily by dendritic cells and macrophages, may affect the production of CXCL9, CXCL10, and CXCL11 by driving IFN-γ secretion [41]. A previous study demonstrated that CXCL9, CXCL10, and CXCL11 levels can predict HIV-1 disease progression during primary HIV-1 infection [42]. Therefore, blocking the CXCL9, CXCL10, and CXCL11/CXCR3 axes shows potential as an approach for decreasing immune activation and exhaustion. Multivariate regression analysis showed that a combination of CDCP1, CXCL11, CST5, SLAMF1, TRANCE, and CD5 could be used to distinguish INRs from IRs. CDCP1, a ligand for CD6 expressed on T cells, acts as a co-stimulatory receptor in the immunological synapse and has been proposed as a molecular target for treating malignancy [43]. As previously described, CXCL11 is associated with HIV-1 disease progression. CST5, which encodes an inhibitor of several cysteine proteases of the cathepsin family, is a candidate tumour suppressor gene [44]. CST5 and TRANCE can suppress the activation and resorption of osteoclasts [45,46], which may be associated with HIV-1-related bone disease. SLAMF1, also known as CD150, plays an important role in immunosuppression of HIV-1 infection. Patients with acute HIV-1 infection show decreased CD150 expression on T cells, which correlates with impaired Th1 cell-mediated responses [47,48]. CD5 has been widely reported to be involved in modulating signalling through antigen receptors in both T and B cells. We found that CD5 levels were lower in INRs than in IRs, which may reflect a low capacity to respond to immune activation.

Persistence of the HIV-1 reservoir is a major obstacle to eradicating HIV-1 and is the underlying cause of immune activation and inflammation [49]. We found that ST1A1, CASP8, SIRT2, AXIN1, STAMBP, CD40, and IL7 in cluster 5 were negatively associated with HIV-1 DNA. ST1A1, a cytosolic sulfotransferase, regulates HIV-1 reverse transcription in monocyte-derived macrophages [50]. CASP8, a caspase protein, is the upstream protease involved in the activation cascade responsible for death receptor-induced cell death [51]. SIRT2 is a member of the sirtuin family, which is comprised of NAD(+)-dependent deacetylases involved in several cellular processes. SIRT2 can regulate NF-κB signalling via deacetylase p65 [52], which may affect HIV-1 latency [53]. AXIN1, a regulator of WNT signalling, plays a negative role in HIV-1 transcription through the WNT signalling pathway under normal cell culture conditions [54]. STAMBP, also known as AMSH, inhibits the release of inflammatory factors by negatively regulating NLRP3 inflammasome destabilization [55]. A previous study reported that structural destabilization of STAMBP leads to loss of function during HIV-1 budding [56]. CD40, also called TNFRSF5, interacts with its ligand CD40L, which contributes to the immunological control of HIV-1 replication by inducing HIV-suppressive chemokines and supporting the production of anti-HIV antibodies and cytotoxic T cells [57]. IL7 is crucial for T cell survival and homeostatic maintenance of peripheral T cells. IL7 significantly enhances HIV-1 proviral reactivation [58]. Although negative correlations were observed between these proteins and HIV-1 DNA, their causal relationship requires further investigation. In addition, we found that ST1A1, CASP8, SIRT2, AXIN1, STAMBP, CD40, and IL7 in cluster 5 were negatively associated with HIV-1 DNA in IRs but not in INRs. HIV-1 DNA is relatively stable in IRs with an optimal immune status. However, INRs are susceptible to opportunistic infections. The weakened immune surveillance of HIV-1 upon reactivation coupled with infection-induced immune activation may impact the stability of the HIV-1 reservoir in INRs. Moreover, compared to IRs, INRs have higher levels of HIV-1 DNA and RNA [59,60].

There were several limitations to this study. First, the mechanisms how IRPs affect T cell immune recovery requires further analysis, as the associations do not necessarily indicate causality. Second, the PCR-based assay in our study overestimated the HIV-1 reservoir size, as it cannot distinguish between replication competent and defective proviral genomes. Finally, because of the lack of samples, we could not assess the functions of T cell subsets, including cytotoxicity and cytokine-secreting abilities. Future studies are needed to reveal the association of aberrant IRPs with T cell functions.

In conclusion, inflammation and T cell dysregulation persist in ART-treated HIV-1-infected individuals. Some IRPs in cluster 7, such as CXCL11, CXCL9, TNF, CXCL10, and IL18, are closely associated with HIV-1 disease progression and immune disorders, irrespective of whether patients received ART treatment. These results may facilitate identification of an effective immune intervention strategy.

## Study approval

This study was approved by the ethics committee of the Fifth Medical Center of Chinese PLA General Hospital (ky-2021-7-6-1), and written informed consent was obtained from all study participants in accordance with the Declaration of Helsinki.

## Data Availability

The datasets generated during and/or analyzed during the current study are available from the corresponding author on reasonable request.
